# Improving the Functional Activities of Curcumin Using Milk Proteins as Nanocarriers

**DOI:** 10.3390/foods9080986

**Published:** 2020-07-24

**Authors:** Soad Taha, Ibrahim El-Sherbiny, Toshiki Enomoto, Aida Salem, Emiko Nagai, Ahmed Askar, Ghada Abady, Mahmoud Abdel-Hamid

**Affiliations:** 1 Dairy Science Department, Faculty of Agriculture, Cairo University, Giza 12613, Egypt; mahmoud.mohamed@agr.cu.edu.eg; 2 Nanomaterials Lab, Center of Material Science (CMS), Zewail City of Science and Technology, 6th of October, Giza 12588, Egypt; ielsherbiny@zewailcity.edu.eg; 3 Department of Food Science, Ishikawa Prefectural University, Nonoichi, Ishikawa 921-8836, Japan; enomoto@ishikawa-pu.ac.jp (T.E.); nagai.e1025@gmail.com (E.N.); 4 Dairy Technology Department, Animal Production Research Institute, Agricultural Research Center, Giza 12618, Egypt; aidas_salem@hotmail.com (A.S.); ghabady@hotmail.com (G.A.); 5 Botany and Microbiology Department, Faculty of Science (Boys), Al-Azhar University, Nasr City, Cairo 11884, Egypt; drahmed_askar@azhar.edu.eg

**Keywords:** curcumin, milk proteins, nanoparticles, antioxidant, anticancer, antimicrobial activities

## Abstract

Curcumin is one of the most common spices worldwide. It has potential benefits, but its poor solubility and bioavailability have restricted its application. To overcome these problems, this study aimed to assess the efficacy of sodium caseinate (SC), α-lactalbumin (α-La), β-lactoglobulin (β-lg), whey protein concentrate (WPC) and whey protein isolate (WPI) as nanocarriers of curcumin. Furthermore, the antioxidant, anticancer and antimicrobial activities of the formed nanoparticles were examined. The physicochemical characteristics of the formed nanoparticles as well as the entrapment efficiency (%) and the in vitro behavior regarding the release of curcumin (%) were examined. The results showed that the formation of curcumin–milk protein nanoparticles enhanced both the entrapment efficiency and the in vitro behavior release of curcumin (%). Cur/β-lg nanoparticles had the highest antioxidant activity, while SC and WPC nanoparticles had the highest anticancer effect. The antimicrobial activity of the formed nanoparticles was much higher compared to curcumin and the native milk proteins.

## 1. Introduction

Curcumin is a bright yellow powder produced from *Curcuma longa* plants. It is the principal curcuminoid of turmeric (*Curcuma longa*), which is used as an herbal supplement, cosmetics ingredient, food flavoring and food coloring [1]. Curcumin has been reported to display several biological activities, such as antioxidant, antibacterial, anti-inflammatory, anti-amyloid and anticancer activities in addition to wound healing and antibiofilm properties [1,2]. However, the practical application of curcumin in functional food formulations is limited due its poor water solubility and bioavailability and rapid degradation under neutral and alkaline pH conditions [3]. Many attempts have been made to enhance the solubility and bioavailability of curcumin, including the use of emulsions, liposomes and nanoparticles [3]. The application of nanotechnology is an important tool to improve the solubility and bioavailability of curcumin [4]. A variety of nanoparticle formulations has been developed to improve the functional characteristics of curcumin [5,6]. In this context, various nanocarriers, generally made of biodegradable substances, such as proteins (including milk proteins), liposomes, carbohydrates and polysaccharides (i.e., chitosan), have been used [7,8,9].

Milk proteins have a specific structural and functional diversity which allows their utilization as agents of encapsulation and for the transport of bioactive molecules. Milk proteins can be used as carriers of hydrophobic molecules or ions, and they are excellent interfacial agents, meaning that they are used in the formation and stabilization of emulsions containing hydrophobic molecules. Furthermore, milk proteins are able to form covalent or electrostatic complexes with molecules of interest and to entrap bioactives through the formation of gels. Furthermore, milk proteins are able to self-assemble or co-assemble to form supra-structures that allow the encapsulation and the transport of a diversity of small molecules [10]. In this context, using β-lg as carrier for curcumin significantly enhanced the water solubility, resistance to pepsin, and pH stability of curcumin [11]. In addition, a complex of whey protein isolate with curcumin increased the solubility of curcumin 180-fold [12]. Furthermore, a whey protein isolate microgel loaded with curcumin had higher antioxidant activity compared to curcumin dissolved in water [13]. The encapsulation of curcumin in camel beta-casein increased the solubility of curcumin 2500-fold. Moreover, the antioxidant activity and cytotoxicity to the human leukemia cell line of curcumin were higher than both camel beta-casein and curcumin [14]. Bovine serum albumin (BSA)–dextran–curcumin nanoparticles exhibited a cellular antioxidant activity in Caco-2 cells that was higher than free curcumin [15].

Consequently, the aim in this study was to evaluate the efficacy of different milk proteins as nanocarriers of curcumin. Furthermore, the antioxidant, anticancer and antimicrobial activities of the developed milk protein–curcumin nanoparticles were assessed.

## 2. Materials and Methods

### 2.1. Materials

Sodium caseinate (SC) was kindly provided by Friesland Campina DMV, Nederland. Whey protein concentrate (WPC 80%) was kindly provided by Onalaska Wisconsin, USA. Whey protein isolate (WPI), α-lactalbumin (*α*-La 97.46% protein) and *ß*-lactoglobulin (*ß*-lg 97.8% protein) were kindly provided by Davisco Foods International, USA. Low molecular weight chitosan (Cs) and sodium tripolyphosphate (TPP) were purchased from Acros Organics, New Jersey, USA. Curcumin (Cur ≥ 96.1% purity) was purchased from Kolorjet Chemicals Pvt Ltd., Mumbai, Maharashtra, India. 1,1-diphenyl-2-picrylhydrazyl (DPPH), tetracycline, amphotericin B, nutrient agar medium and Czapek’s Dox agar medium (for antifungal activity) were purchased from Sigma Chemical Company St. Louis, MO, USA. Human hepatocellular carcinoma (HepG2), human breast adenocarcinoma (MCF-7), Gram-positive (*Bacillus subtilis* ATCC 6633, *Staphylococcus aureus* ATCC 29213) and Gram-negative bacteria (*Pseudomonas aeruginosa* ATCC 27853, *Escherichia coli* ATCC 25922) and *Candida albicans* (ATCC10231) were obtained from the American Type Culture Collection (ATCC) Manassas, Virginia, USA. Roswell Park Memorial Institute (RPMI) 1640 medium was purchased from Genetix Biotech, Asia Pvt. Ltd., India. 3-(4,5-Dimethyl-2-thiazolyl)-2,5-diphenyl-2H-tetrazolium·bromide (MTT) was purchased from SERVA Electrophoresis GmbH, Heidelberg, Germany. Phosphate buffered saline (PBS) tablets were purchased from Fountain Pkwy, Solon, USA. All other chemicals used were of analytical grade.

### 2.2. Experimental Procedures and Methods of Analysis

#### 2.2.1. Preparation and Characterization of Milk Protein–Chitosan Nanocomposite

Nanocomposites of milk protein–chitosan were prepared by the ionotropic gelation method [16]. Briefly, 1 mL of protein solution (10%) was added to 50 mL chitosan solution (0.2% prepared in 1% acetic acid) under mild stirring. Then, 15 mL sodium tripolyphosphate solution (TPP 0.5%) was added drop-wise to the mixture. The overall mixture was stirred for 30 min at 25 °C. The resulting particles were collected by centrifugation (Avanti j-301 Beckman, CA, USA) at 25,000 rpm for 30 min at 4 °C, washed twice with distilled water and then freeze-dried (Labconco freeze dryer, USA).

#### 2.2.2. Preparation of Curcumin-Loaded Milk Proteins

Curcumin solution was prepared separately by dissolving curcumin (100 mg) in ethanol until the solution became clear; then, the solution was added to the milk protein–chitosan nanosuspension (without freeze drying) and stirred for 30 min at 25 °C, followed by centrifugation at 25,000 rpm for 30 min at 4 °C. The resulting curcumin-loaded nanoparticles (NPs) were washed twice with distilled water and then freeze-dried.

### 2.3. Characterizations of Nanoparticles

#### 2.3.1. Particle Size and Zeta Potential Measurements

The size distribution and zeta potential of the resultant nanoparticles were determined using a zetasizer (Nano-ZS, Malvern, UK) [17]. The measurements were performed at 25 °C after the appropriate dilution of samples with water (1:400). The zeta potential was measured using a disposable zeta cuvette.

#### 2.3.2. Physicochemical Characterization

The Fourier-transform infrared spectroscopy (FTIR) spectra of native and curcumin-loaded milk proteins nanoparticles were examined by an FTIR spectrometer (NICOLET iS10, Thermo Scientific Inc., USA) in the attenuated total reflection (ATR) mode [18]. The spectra were recorded in the wavenumber range of 600–4000 cm^−1^.

The morphology of curcumin-loaded milk protein nanoparticles was imaged using transmission electron microscopy (TEM) (JEOL, JEM1400, Japan) according to Swed et al. [19]. Drops of aqueous suspension of nanoparticles suspended in distilled water were deposited in carbon-coated copper grids and then dried at room temperature before examination.

### 2.4. Determination of the Entrapment Efficiency (EE %) and in vitro Release %

The entrapment efficiency (EE %) was determined according to Sadeghi et al. [20]. The amount of curcumin encapsulated into the developed nanoformulations was obtained by determining the difference between the total amount of curcumin added initially into the preparation medium and the amount which remained in the supernatant after centrifugation. The free curcumin present in the supernatant was determined spectrophotometrically at 426 nm using a UV–Vis spectrophotometer (Evolution UV 600, Thermo Scientific, USA). The EE% was calculated using the following equation:

$$\mathrm{EE}\left(\%\right)=\frac{\mathrm{Initial}\;\mathrm{amount}\;\mathrm{of}\;\mathrm{curcumin}-\mathrm{free}\;\mathrm{curcumin}\;\mathrm{in}\;\mathrm{supernatant}}{\mathrm{Initial}\;\mathrm{amount}\;\mathrm{of}\;\mathrm{curcumin}}\times 100$$

The method of Teng et al. [21] was adapted to determine the in vitro release percentage of curcumin from the nanoparticles. Curcumin-loaded milk protein nanoparticles (5 mg) were mixed with 2 mL of PBS at pH 7.4. The blend was placed into a dialysis bag (MW cutoff 10 kDa) which was then put in 45 mL falcon tubes containing 20 mL of PBS with 0.5% Tween 20. The falcon tubes were incubated in a shaking bath (Thermo Scientific, MaxQ4450, USA) at 37 °C under constant shaking at 110 rpm. Afterwards, a 1 mL aliquot of the PBS medium was withdrawn at specific intervals of 0.25, 0.50, 1, 2, 4, 6, 24 and 48 h and replaced by a fresh medium. The absorbance was measured at 426 nm, which was converted to the mass of released curcumin using a linear standard curve (R^2^ = 0.9976). The concentration of curcumin at different time intervals was monitored, and then calculated using the following equation:

$$C_{n}=C_{n\;means}+A/V\cdot \overset{n-1}{\underset{S=1}{\sum }}C_{s\;means}$$

where *C_n_* is the expected theoretical sample concentration, *C_n means_* is the measured concentration, *A* is the volume of withdrawn aliquot (20 mL), *V* is the volume of the dissolution medium (2 mL), *n−1*(20-1) is the total volume of all the previously withdrawn samples before the currently measured sample and *C_s_* is the total concentration of all previously measured samples before the currently measured sample. The in vitro release % of curcumin from the resultant nanocarriers was measured in triplicate, and the cumulative release percentage was plotted against time.

### 2.5. Antioxidant Activity

The antioxidant activity (radical scavenging activity %) was measured using DPPH (1,1-diphenyl-2-picrylhydrazyl) [22]. In brief, 1.5 mL of the tested materials at concentrations of 1.25, 2.5 and 5 mg/mL was mixed with 1.5 mL of 0.1 mM DPPH in ethanol. The mixtures were vortexed and incubated in the dark for 30 min at room temperature, and then the absorbance was measured at 517 nm.

$$\mathrm{Radical}\;\mathrm{scavenging}\;\mathrm{activity}\left(\%\right)=\left(A_{0}-A_{1}/A_{0}\right)\times 100$$

where A_0_ is the absorbance of the control without samples (DPPH solution) and A_1_ is the absorbance of the tested materials.

### 2.6. Anticancer Activity

The cytotoxicity of the tested materials on HepG2 and MCF-7 cell lines was determined by MTT assay [23]. The cells were plated (1 × 10^5^ cells/mL) in 96 well plates and incubated at 37 °C for 24 h to develop a complete monolayer sheet. The plates were washed with phosphate buffered saline (pH 7.4) and then incubated at 37 °C for 24 h in the presence of 0.1 mL of the tested material solutions (2.5, 5 and10 mg/mL) in Roswell Park Memorial Institute (RPMI) medium (maintenance medium). Then, 20 µL/well of MTT in buffered saline solution (5 mg/mL) was added, and the plates were incubated at 37 °C in a CO_2_ incubator for 1–5 h to allow the MTT to be metabolized, forming formazan. The optical density at 560 nm and 620 nm (subtract background) was measured. The effect of the tested materials on the proliferation of human cancer cells is expressed as the cell viability % using the following formula:

Cell viability(%)=absorbance of treated cells/absorbance of control cells×100

### 2.7. Antimicrobial Activity

The antimicrobial activity of all tested materials were examined against Gram-positive (*Bacillus subtilis* and *Staphylococcus aureus*) and Gram-negative bacteria (*Pseudomonas aeruginosa* and *Escherichia coli*) as well as *Candida albicans* using the agar well diffusion method [24]. All strains (individually) were grown in 10 mL of nutrient broth and adjusted to a count of 10^8^ cells/mL for bacteria or 10^5^ cells/mL for fungi using a spectrophotometer. Tested strains (100 μL) were added to the growth medium and allowed to solidify. One milliliter of each tested material (500 µg/mL) was added to each well. The plates were incubated at 37 °C for 24 h for bacteria and at 27 °C for 72 h for fungi. After incubation, the growth of microorganisms was observed. The antimicrobial activity was evaluated by measuring the diameter of the zone of transparent inhibition against tested microorganisms. Tetracycline was used as a standard antibacterial drug, while amphotericin B was used as a standard antifungal drug.

### 2.8. Statistical Analysis

A randomized complete block design with two factors (factor A: concentration. and factor B: treatments) was employed. For the other parameters, we used a randomized complete block design with one factor, with three replications for each parameter. The treatment means were compared by the least significant difference (L.S.D.) [25].

## 3. Results and Discussion

### 3.1. Characterizations of Nanoparticles

#### 3.1.1. Particle Size

The data in Table 1 show that the average particle size of the milk protein–chitosan nanocomposite ranged from 275.33 to 334.90 nm with non-significant differences between all treatments except for Cs/WPI NPs. After loading curcumin, the average particle sizes decreased in the case of casein, α-La and β-Lg nanoparticles with non-significant differences and reached 278.10, 290.83 and 274.80 nm, respectively, while the sizes significantly increased in the case of WPI and WPC nanoparticles, reaching 462.80 and 439.90 nm, respectively, which may be due to the formation of large aggregates via nonspecific interactions [26]. All nanoparticles—either without or with curcumin—are the under nano-scale, which agreed with the data reported by Singh et al. [27] and Arroyo-Maya et al. [28].

#### 3.1.2. Zeta Potential

Zeta potential values ranged from −11.13 to −14.03 mV (pH 5.3) for milk protein–chitosan nanocomposites, with the exception of Cs-WPI nanocomposite, where the value was −17.30 mV (pH 4.3), with significant differences with other treatments. In case of curcumin-loaded milk protein nanoparticles, the zeta potential ranged from −12.63 to −19.5 (pH 5.2–5.42), while it changed to be highly positive for Cur-Cs/WPC, at +27.73 mV (pH 4.2). Significant differences were noticed between all curcumin NPs (Table 1). These findings are in agreement with Awad et al. [29] and Sangeetha et al. [17].

#### 3.1.3. Fourier-Transform Infrared Spectroscopy (FTIR)

**Figure 1:**
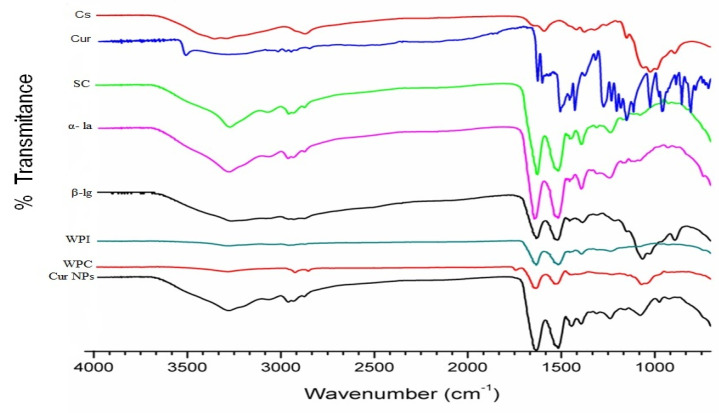
Fourier-transform infrared (FTIR) spectra of chitosan (Cs), curcumin (Cur), Sod. caseinate (SC), α-lactalbumin (α-La), β-lactoglobulin (β-lg), whey protein isolate (WPI), whey protein concentrate (WPC) and curcumin loaded nanoparticles (Cur NPs).

FTIR spectra of native and curcumin-loaded nanoparticles explain any structural differences between pure compounds and the different developed nanoparticle systems. As shown in Figure 1, the spectrum of native sodium caseinate (SC) had four characteristic peaks at 3273, 2960, 1635 and 1515 cm^−1^ attributed to NH_2_/OH, C-H stretching, C=O stretching and NH_2_ bending, respectively, while for α-La, the four characteristic peaks of the FTIR spectra appeared at 3273, 2960, 1650 and 1500 cm^−1^, attributed to NH_2_/OH, CH stretching, C=O and NH_2_ bending, respectively. Furthermore, the FTIR spectra of native β-Lg showed four characteristic absorbance peaks at 3271, 2957, 1633 and 1515 cm^−1^, attributed to NH_2_/OH, CH stretching, NH/C=O and NH_2_ bending of the amide group respectively. With respect to WPC and WPI, similar results were found in terms of their FTIR spectra, which exhibited three characteristic absorbance peaks at 2957, 1633 and 1532 cm^−1^, which correspond to CH stretching, NH/C=O and N=O, respectively. It is also clear that the spectrum of chitosan showed three characteristic absorbance peaks. One peak at 3354 cm^−1^ corresponds to the combined peaks of NH and OH groups in chitosan, while the peaks at 1592 and 1377 cm^−1^ correspond to the N-H bending of the amide group (N-acetylated residues) and symmetrical CH_3_, respectively. The FTIR spectrum of curcumin exhibited three strong peaks at 3272, 1505 and 1625 cm^−1^, attributed to CH/NH_2_, OH and N=O, respectively (Figure 1). The characteristic absorbance peak at 3507 cm^−1^, which corresponds to the CH stretching vibration of curcumin, disappeared after encapsulation. Furthermore, there was a shift in the band peak from 1625 and 1505 to 1633 and 1520 cm^−1^ after nanoparticles were loaded with curcumin (Cur-NPs). These peaks shifted slightly toward a higher wavenumber, and new absorption bands appeared (C=O and N=O). This may be due to the structural changes of curcumin during the encapsulation process. In general, it was clear from these results that NPs were formed due to the interaction between the carboxyl group (–COO–) of the protein and amino groups of chitosan. In addition, there was an interaction between milk protein–chitosan nanocomposites and curcumin; the same spectra were found for curcumin milk protein–chitosan nanoparticles with slight shifting (Figure 1), and this may be a result of the electrostatic interactions during the formation of curcumin-loaded milk protein nanoparticles (Cur-NPs). These results are in line with those of Udompornmongkol and Chiang [30].

#### 3.1.4. Transmission Electron Microscopy (TEM)

**Figure 2:**
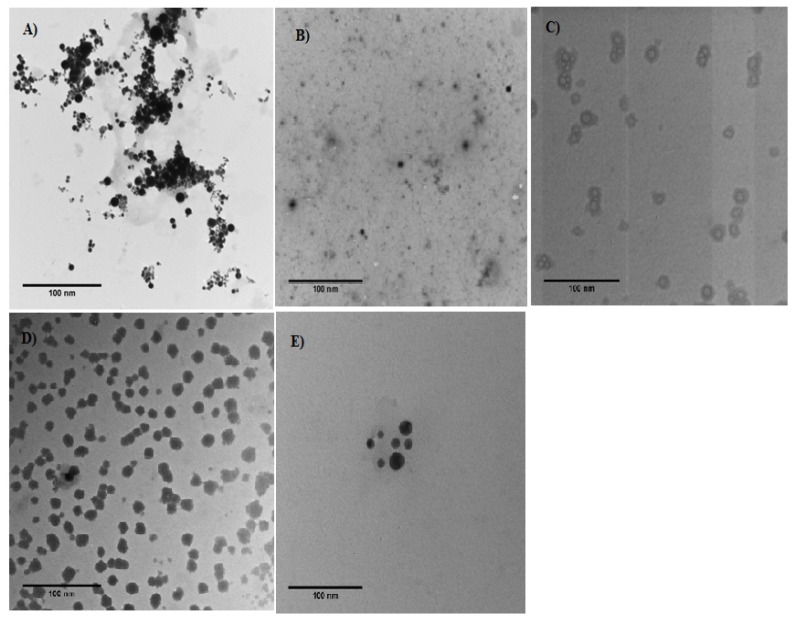
Transmission electron microscope (TEM) images of curcumin-loaded milk protein nanoparticles. (**A**) Cur-SC NPs, (**B**) Cur-α-La NPs, (**C**) Cur-β-lg NPs, (**D**) Cur-WPI NPs, (**E**) Cur-WPC NPs.

Figure 2 shows the morphological characteristics of the prepared nanoparticles from the various treatments. The prepared nanoparticles were regular and spherical in shape, with sizes under the nano-scale. The average size matched that observed in the dynamic light scattering DLS values. The loading of curcumin resulted in an increase in the particle size, particularly with WPI and WPC, which matched the DLS values.

### 3.2. Entrapment (Encapsulation) Efficiency (EE %)

The EE% ranged from 72.27–77.27% with significant differences between all treatments (Table 1). Both Cur-Cs/WPI NPs and Cur-Cs/WPC NPs had the highest EE% values, at 76.30% and 77.27%, respectively, while the lowest EE% was achieved for SC, at 72.27%, which may be due to the presence of more binding sites on WPC and WPI than other types of proteins. These results are in line with those reported by Chen and Subirade [31], Pan et al. [32] and El-Sayed et al. [33].

### 3.3. The In Vitro Behavior Release of Curcumin (%)

**Figure 3:**
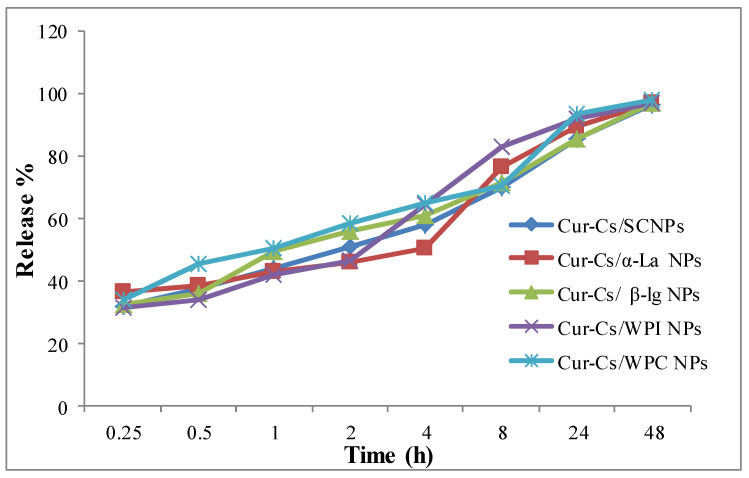
In vitro release profiles of curcumin from curcumin–milk protein nanoparticles.

In general, the in vitro behavior release of curcumin (%) increased gradually in all treatments as the experimental time increased, and this was the highest for curcumin-loaded casein nanoparticles (Figure 3). In the first 2 h, about 50% of the encapsulated curcumin was released from casein, α-la and WPC, while this value ranged from 31.45% to 36.21% in the first 15 min and gradually increased with time to reach 96.33–97.83% after 48 h. The obtained results indicated that milk proteins can be successfully considered to be carriers for curcumin. The release of curcumin was controlled by its dissociation from the porous polymer matrix and particle size; a smaller size led to a faster release due to the increase in the surface area [34].

### 3.4. Antioxidant Activity (%)

As shown in Table 2, the antioxidant activity was dependent on concentration. Furthermore, the antioxidant activity of all native proteins was significantly lower than that of both of chitosan or curcumin, except for both β-lg and WPI, whose antioxidant activities were slightly higher or close to curcumin. The antioxidant activity ranged from 46.12% to 60.32% and 41.60% to 62.13% for chitosan and curcumin, respectively, while it ranged from 36.30% to 43.70% for native milk proteins at the level of 1.25 mg/mL and from 52.80% to 61.03% at the level of 5 mg/mL. It is obvious that the antioxidant activity of all curcumin-loaded milk protein nanoparticles is significantly higher than that of chitosan, curcumin and all native proteins or nanocomposites with chitosan. The antioxidant activity of curcumin–milk protein nanoparticles can be arranged in descending order as follows: Cur/β-lg > Cur/WPI > Cur/SC > Cur α-la > Cur/WPC. These results are consistent with Yi et al. [35], who reported that the DPPH scavenging activity of curcumin encapsulated with milk proteins such as α-La was dramatically enhanced due to the increased water solubility and greater surface area, which facilitated the interaction between curcumin and radicals. Previous studies have demonstrated that WPI alone exhibits antioxidant activity, while the WPI–curcumin microparticles showed higher antioxidant activity than curcumin or native WPI [36]. Furthermore, the use of β-casein as a carrier of curcumin enhanced its solubility and as a result improved the functional activities of curcumin nanoparticles [14].

### 3.5. Anticancer Activity

The data presented in Table 3 and Table 4 show the anticancer activity of all tested materials against human hepatocarcinoma (HepG2) and human breast carcinoma cells (MCF-7). It is generally evident that as the concentration of the tested materials increased and that the anticancer activity significantly increased [37].

As for HepG2 cancer cells, as shown in Table 3, it is evident that the anticancer activity of curcumin is considerably greater than that of chitosan, which is in agreement with Senft et al. [38] and Gupta et al. [39] who reported that curcumin has a higher toxic effect on tumor cells owing to the capability of curcumin to interfere with several biochemical pathways involved in the proliferation and survival of cancer cells. Furthermore, Bouhenna et al. [40] stated that the anticancer activity of chitosan may be due to the interactions between the charged groups of chitosan molecules and tumor cells.

The anticancer activity of the native milk proteins ranged from 17.55% to 36.75% (at the level of 2.5 mg/mL). Native β-lg had the lowest effect, while α-la tended to have the highest effect. The mechanism of action of WP on tumor development is due to increased concentrations of tissue glutathione, which detoxifies free radicals and improves the immune response [41]. Additionally, the anticancer activity of curcumin-loaded milk protein nanoparticles is significantly higher than that of the native milk proteins or chitosan–milk protein nanocomposite. The inhibitory activity % of SC and WPC in all forms was the highest compared to the other tested materials, which confirmed the work of Pan et al. [42], who reported that the entrapment of curcumin in casein nanoparticles resulted in higher antioxidant activity and cytotoxicity against cancer cells compared to free curcumin. This may be due to the antimutagenic properties of the casein structure, which can control the mutagen rather than its amino acid composition. Furthermore, Krissansen [43] noted that whey protein concentrate could play a potential role in cancer treatments as it increased baicalein’s cytotoxicity to the HepG2 cell line.

As regards the MCF-7 cancer cells, as previously mentioned, the anticancer activity of all tested materials increased significantly as their concentration increased. Both chitosan and curcumin had almost the same anticancer activity (Table 4). It is apparent that native milk proteins (SC and α-La) had almost the same anticancer activity (at the level of 10 mg/mL) as both chitosan and curcumin, and they also had the highest effect compared to the other milk proteins. The inhibition % of the native milk proteins ranged from 16.63% (WPI) to 30.27% (α-la) with significant differences between the native proteins at the level of 2.5 mg/mL, while it increased to 76.30% (WPC) and 93.25% (α-la) at the level of 10 mg/mL. The inhibitory effect of whey proteins may be due to its content of sulfur amino acids which enhanced the glutathione bioavailability and reduced oxidative stress, leading to cancer prevention [44]. The anticancer activity of the milk protein–chitosan nanocomposite was higher than native proteins. These findings also implied that curcumin-loaded milk protein nanoparticles showed the highest anticancer activity relative to all the tested materials. These results are in line with Adahoun et al. [45], who reported that curcumin NPs had a much stronger antiproliferative impact on cancer cells compared to native curcumin due to their ability to inhibit specific molecular signaling pathways involved in carcinogenesis. Tabatabaei et al. [46] proved that curcumin-loaded poly(lactide-co-glycolide)-poly(ethylene glycol) (PLGA-PEG) has more cytotoxic effects on the MCF-7 breast cancer cell line due to the enhancement of its water solubility as well as its bioavailability and functionality compared to curcumin.

### 3.6. Antimicrobial Activity

As shown in Table 5, the antibacterial effect of curcumin was slightly higher, with non-significant differences compared to chitosan. The inhibition zone ranged from 16.00 to 23.00 mm for curcumin, while it ranged from 14.00 to 21.00 mm for chitosan. Furthermore, it is notable that *B.subtilis* was more sensitive to curcumin and chitosan compared to the other strains. Additionally, all tested materials displayed variable antibacterial activity. All native forms of milk proteins had almost no effect on all tested strains, except SC; its antibacterial effect was higher than that of both of chitosan or curcumin. Its inhibition zone ranged from 15.00 to 26.00 mm. The inhibitory effect of curcumin may be due to cell membrane damage causing membrane permeabilization [47], while the key mechanism of chitosan is due to its cationic nature and the electrostatic interaction between positively charged chitosan groups and negatively charged sites on the microbial cell and its penetration into the bacteria cell wall, which is linked to the microorganism DNA inhibiting the transcription and consequently the translation process [48].

The formation of nanoparticles of milk proteins with chitosan or curcumin (in most cases) significantly enhanced the inhibitory effect compared to chitosan, curcumin and native milk proteins. It is also noticeable that all curcumin-loaded milk protein nanoparticles had increased activity against *E. coli* and *B. subtilis*. The inhibition zone ranged from 24 to 31 mm and from 28 to 33 mm, respectively, as compared to the other tested strains. *P. aeruginosa* and *Staph. aureus* were less sensitive to the tested curcumin-loaded nanoparticles (Table 5). The inhibition zone ranged from 20 mm (Cur-Cs/α-La NPs and Cur-Cs/WPI NPs) to 22 mm (Cur-Cs/βlgNPs and Cur-Cs/WPC NPs) for *Staph. aureus*, and from 20 mm (Cur-Cs/WPC NPs) to 28 mm (Cur-Cs/α-La NPs) for *P. aeruginosa*. These results are in line with those of Deka et al. [49] who reported that the water solubility and antimicrobial activity of curcumin were significantly improved by curcumin nanoparticle formation compared to curcumin alone.

*Candida albicans* is a major fungal pathogen of humans, affecting millions of people and causing death worldwide [50]. The antifungal activity of curcumin is significantly higher than that of chitosan, at 21 vs. 12 mm (Table 5). The antifungal effect of curcumin against *C. albicans* was due to the disruption of the cell wall [51]; for chitosan, the positive charge of chitosan can interact with the negatively charged microbial cell surface and disrupt the anion–cation balance, thereby exerting an inhibitory effect [52].

Only native SC and α-La had an antifungal effect similar to chitosan, and this was significantly lower than curcumin (Table 5). The formation of nanocomposite and curcumin-loaded milk protein nanoparticles enhanced the antifungal effect more than the native forms. Cur-Cs/SCNPs were most effective against *C. albicans* compared to the other tested materials. The diameter of the inhibition zone ranged from 21 mm (Cur-Cs/WPINPs) to 28 mm (Cur-Cs/SCNPs). It was also remarkable that the inhibitory effect of the tested nanoparticles in most cases was higher than that of the used standard antibiotics, which agreed with the results of Paul et al. [53], who reported that curcumin–silver nanoparticles displayed antifungal activity against different isolates of candida species as compared to curcumin and AgNO_3_ solutions. The inhibitory effect of the nanoparticles can be arranged in descending order as follows: Cur/SC > Cur/α-La > Cur/WPC > Cur/βlg > Cur/WPI.

## 4. Conclusions

The obtained results revealed that all milk proteins used had a proven efficiency as nanocarriers for curcumin. All curcumin-loaded milk protein nanoparticles exhibited antioxidant and anticancer activity on both HepG2 and MCF-7 cell lines due to the enhanced solubility and bioavailability of curcumin. This effect was dose-dependent. Curcumin nanoparticles with α-lactalbumin showed the highest antioxidant activity at a concentration of 2.5 mg/mL. Furthermore, curcumin nanoparticles with WPI had the highest anticancer activity against HepG2 cell line, while curcumin nanoparticles with β-lactoglbulin exhibited the highest anticancer activity against MCF-7 cell line. Moreover, all nanoparticles displayed antibacterial and anticandida effects. It is worth noting that curcumin nanoparticles with β-lactoglbulin and WPC showed the highest antimicrobial activity against all tested strains. These results allow and encourage the use of these compounds in the food and medical sectors.

## Figures and Tables

**Table 1:** Particle size, zeta potential and entrapment efficiency (EE %) of curcumin-loaded milk protein nanoparticles.

Treatments	Particle Size (nm)	Zeta Potential	EE%	pH
Cs/SCNPs	328.67 ^b^ ± 47.55	−12.73 ^cd^ ± 0.87	-	5.00
Cs/α-LaNPs	334.90 ^b^ ± 5.84	−12.70 ^cd^ ± 0.44	-	5.21
Cs/β-lgNPs	310.53 ^bc^ ±13.17	−11.13 ^c^ ± 0.64	-	5.00
Cs/WPI NPs	275.33 ^c^ ± 30.89	17.30 ^b^ ± 1.90	-	4.30
Cs/WPC NPs	318.17 ^bc^ ± 33.98	−14.03 ^d^ ± 1.21	-	5.30
Cur-Cs/SCNPs	278.10 ^c^ ± 16.89	−12.63 ^cd^ ± 2.11	72.27 ^e^ ± 0.13	5.20
Cur-Cs/α-La NPs	290.83 ^bc^ ± 47.17	−19.50 ^e^ ± 0.17	74.67 ^c^ ± 0.06	5.31
Cur-Cs/β-lg NPs	274.80 ^c^ ± 8.84	−14.83 ^d^ ± 2.58	73.97 ^d^ ± 0.12	5.30
Cur-Cs/WPI NPs	462.80 ^a^ ± 6.15	−13.57 ^d^ ± 3.23	76.30 ^b^ ± 0.10	5.42
Cur-Cs/WPC NPs	439.90 ^a^ ± 2.95	27.73 ^a^ ± 0.81	77.27 ^a^ ± 0.06	4.20

Different superscripts (a–e) in the same column are significantly different (*p* ˂ 0.05). SCNPs: sodium caseinate nanoparticles; WPI: whey protein isolate; WPC: whey protein concentrate; NPs: nanoparticles.

**Table 2:** Antioxidant activity (radical scavenging activity %) of different concentrations of native milk proteins, chitosan–milk protein nanocomposite and curcumin-loaded milk protein nanoparticles.

Treatments	Concentration (mg/mL)			Mean
	2.5	5	10	
Chitosan	46.12 ± 2.9	54.08 ± 3.9	60.32 ± 6.7	53.51 ± 4.5
Curcumin	41.60 ± 3.5	54.02 ± 4.0	62.13 ± 2.9	52.58 ± 3.5
SC	39.18 ± 3.0	42.75 ± 3.3	52.28 ± 3.7	44.74 ± 3.3
CS/SCNPs	39.97 ± 2.7	50.48 ± 2.7	66.90 ± 3.5	52.45 ± 3.0
Cur-Cs/SCNPs	49.17 ± 4.4	60.61 ± 4.4	67.54 ± 3.5	59.11 ± 4.1
α-La	36.15 ± 1.7	48.38 ± 3.6	56.55 ± 2.1	47.03 ± 2.5
CS/α-LaNPs	47.06 ± 2.8	56.90 ± 2.5	62.51 ± 3.0	55.49 ± 2.7
Cur-CS/α-La NPs	53.82 ± 2.0	59.35 ± 2.9	66.44 ± 1.9	59.87 ± 2.3
β-lg	43.70 ± 2.9	53.51 ± 2.0	60.49 ± 2.8	52.57 ± 2.6
CS/βlgNPs	46.86 ± 3.1	54.14 ± 3.3	65.90 ± 1.2	55.63 ± 2.5
Cur-Cs/βlg NPs	48.08 ± 2.8	61.62 ± 2.9	69.05 ± 3.5	59.58 ± 3.1
WPI	43.26 ± 3.1	50.52 ± 6.8	60.92 ± 1.9	51.57 ± 3.9
Cs/WPI NPs	51.10 ± 3.8	55.10 ± 2.4	66.49 ± 2.5	57.56 ± 2.9
Cur-Cs/WPINPs	50.11 ± 3.3	55.08 ± 1.6	68.57 ± 2.5	57.92 ± 2.5
WPC	36.30 ± 2.0	52.44 ± 2.4	61.03 ± 4.8	49.92 ± 3.1
Cs/WPC NPs	47.03 ± 3.9	57.26 ± 2.9	65.40 ± 2.7	56.56 ± 3.2
Cur-Cs/WPCNPs	49.03 ± 4.2	56.20 ± 3.1	63.30 ± 2.6	56.18 ± 3.3
Mean	45.21 ± 3.1	54.26 ± 3.2	63.28 ± 3.0	

Least significant difference (L.S.D.) value at 0.05: concentrations: 0.72, treatments: 1.72, interaction: 2.98.

**Table 3:** Anticancer activity of different concentrations of native proteins, chitosan–milk protein nanocomposite and curcumin-loaded milk protein nanoparticles against HepG2 cell lines.

Treatments	Anticancer Activity %/Concentrations (mg/mL)			Mean
	2.5	5	10	
Chitosan	34.97 ± 1.03	73.35 ± 2.25	91.79 ± 1.96	66.70 ± 1.75
Curcumin	54.06 ± 1.94	81.08 ± 2.32	95.07 ± 2.93	76.74 ± 2.40
SC	33.71 ± 1.29	71.33 ± 2.12	93.05 ± 2.95	66.03 ± 2.12
CS/SCNPs	40.4±3.35	82.95±3.35	95.29±1.71	72.88±2.80
Cur-Cs/SCNPs	46.49±2.64	86.36±2.64	97.31±2.04	76.72±2.44
α-La	36.75±1.75	70.70±2.40	92.04±1.36	66.50±1.84
CS/α-LaNPs	38.23±1.77	76.67 ± 1.43	94.11 ± 1.89	69.67 ± 1.70
Cur-Cs/α-La NPs	45.9 ± 1.40	81.79 ± 1.19	96.80 ± 3.30	74.83 ± 1.96
βlg	17.55 ± 2.55	48.36 ± 4.64	83.58 ± 2.42	49.83 ± 3.20
CS/βlgNPs	40.88 ± 2.12	70.84 ± 2.56	92.55 ± 2.45	68.09 ± 2.38
Cur-Cs/βlg NPs	43.27 ± 1.73	85.54 ± 1.37	95.80 ± 2.20	74.87 ± 1.77
WPI	18.59 ± 1.41	53.91 ± 1.19	76.64 ± 1.66	49.71 ± 1.42
CS/WPINPs	43.03 ± 1.53	81.69 ± 1.91	93.81 ± 1.19	72.84 ± 1.54
Cur-Cs/WPINPs	49.9 ± 1.10	82.20 ± 2.79	96.31 ± 2.81	76.14 ± 2.23
WPC	30.63 ± 2.37	81.29 ± 3.71	93.68 ± 2.18	68.53 ± 2.75
Cs/WPC NPs	42.74 ± 2.26	82.53 ± 1.07	95.27 ± 3.23	73.51 ± 2.19
Cur-Cs/WPCNPs	48.68 ± 4.32	80.36 ± 3.06	98.07 ± 3.07	75.70 ± 3.48
Mean	39.16 ± 2.03	75.94 ± 2.35	93.01 ± 2.31	

L.S.D. value at 0.05: concentrations: 0.69, treatments: 1.63, interaction: 2.83.

**Table 4:** Anticancer activity of different concentrations of native proteins, chitosan–milk protein nanocomposite and curcumin-loaded milk protein nanoparticles against MCF7 cell lines.

Treatments	Anticancer Activity (%)/Concentrations (mg/mL)			
	2.5	5	10	Mean
Chitosan	33.22 ± 1.7	67.21 ± 1.6	92.21 ± 2.8	64.21 ± 2.0
Curcumin	33.4 ± 1.7	73.27 ± 2.0	93.52 ± 2.0	66.73 ± 1.9
SC	25.25 ± 2.1	71.27 ± 2.6	92.93 ± 2.1	63.15 ± 2.3
CS/SCNPs	35.86 ± 3.9	75.91 ± 3.9	93.33 ± 1.8	68.37 ± 3.7
Cur-Cs/SCNPs	50.84 ± 2.9	78.15 ± 2.9	96.88 ± 5.5	75.29 ± 3.7
α-La	30.27 ± 2.8	71.27 ± 2.0	93.25 ± 1.8	64.93 ± 2.2
CS/α-La NPs	41.61 ± 3.1	75.13 ± 4.1	94.78 ± 3.7	70.51 ± 3.6
Cur-Cs/α-La NPs	51.26 ± 2.3	77.18 ± 2.2	98.31 ± 1.8	75.58 ± 2.1
βlg	19.24 ± 1.8	50.98 ± 3.1	85.37 ± 4.2	51.86 ± 3.0
CS/βlgNPs	44.08 ± 2.9	61.58 ± 2.5	91.93 ± 2.1	65.86 ± 2.5
Cur-Cs/βlg NPs	53.63 ± 2.6	74.61 ± 3.4	98.47 ± 3.5	75.57 ± 3.5
WPI	16.63 ± 2.5	48.80 ± 4.1	83.17 ± 2.8	49.53 ± 3.1
CS/WPINPs	41.65 ± 2.4	74.78 ± 3.8	94.2 ± 1.3	70.21 ± 2.5
Cur-Cs/WPINPs	52.43 ± 2.8	77.87 ± 5.9	98.48 ± 5.5	76.26 ± 4.7
WPC	17.87 ± 2.1	62.24 ± 2.6	76.30 ± 3.0	52.14 ± 2.6
Cs/WPC NPs	33.26 ± 2.2	74.79 ± 3.3	93.86 ± 2.3	67.30 ± 2.6
Cur-Cs/WPCNPs	50.03 ± 1.5	75.96 ± 2.9	98.06 ± 2.5	74.68 ± 2.3
Mean	37.09 ± 2.4	70.06 ± 3.2	89.12 ± 2.9	

L.S.D. value at 0.05: concentrations: 1.15, treatments: 2.73, interaction: 4.7.

**Table 5:** Antimicrobial activity of native proteins, chitosan–milk protein nanocomposite and curcumin-loaded milk protein nanoparticles.

Treatments	Inhibition Zone (mm)				
	*E. Coli*	*Staph. aureus*	*B. subtilis*	*P. aeruginoas*	*C. albicans*
Chitosan	15 ^g^ ± 4	19 ^bcd^ ± 2	21 ^fg^ ± 3	14 ^f^ ± 4	12 ^e^ ± 2
Curcumin	16 ^fg^ ± 3	20 ^bc^ ± 3	23 ^ef^ ± 2	16 ^ef^ ± 3	21 ^bc^ ± 3
SC	19 ^efg^ ± 2	25 ^a^ ± 2	26 ^cde^ ± 4	15 ^f^ ± 3	14 ^e^ ± 2
CS/SCNPs	22 ^cde^ ± 3	17 ^cde^ ± 3	28 ^bcd^ ± 3	20 ^cde^ ± 3	20 ^cd^ ± 4
Cur-Cs/SCNPs	27 ^ab^ ± 3	21 ^b^ ± 4	31 ^ab^ ± 4	25 ^ab^ ± 4	28 ^a^ ± 3
α-La	0 ^h^ ± 0	0 ^f^ ± 0	0 ^h^ ± 0	0 ^g^ ± 0	20 ^cd^ ± 3
CS/α-LaNPs	20 ^def^ ± 3	15 ^e^ ± 4	25 ^def^ ± 3	25 ^ab^ ± 2	22 ^bc^ ± 3
Cur-Cs/α-La NPs	24 ^bcd^ ± 3	20 ^bc^ ± 3	30 ^abc^ ± 3	28 ^a^ ± 3	25 ^ab^ ± 2
Βlg	0 ^h^ ± 0	0 ^f^ ± 0	0 ^h^ ± 0	0 ^g^ ± 0	0 ^f^ ± 0
CS/βlgNPs	25 ^bc^ ± 2	19 ^bcd^ ± 2	30 ^abc^ ± 3	24 ^abc^ ± 3	16 ^de^ ± 3
Cur-CS/βlgNPs	30 ^a^ ± 3	22 ^ab^ ± 3	33 ^a^ ± 2	26 ^ab^ ± 3	21 ^bc^ ± 3
WPI	0 ^h^ ± 0	0 ^f^ ± 0	0 ^h^ ± 0	0 ^g^ ± 0	0 ^f^ ± 0
Cs/WPI NPs	20 ^def^ ± 3	14 ^e^ ± 3	28 ^bcd^ ± 3	15 ^f^ ± 2	16 ^de^ ± 3
Cur-Cs/WPI NPs	28 ^ab^ ± 3	20 ^bc^ ± 3	32 ^ab^ ± 3	22 ^bcd^ ± 3	21 ^bc^ ± 3
WPC	0 ^h^ ± 0	0 ^f^ ± 0	16 ^g^ ± 1	0 ^g^ ± 0	0 ^f^ ± 0
Cs/WPC NPs	24 ^b-d^ ± 3	16 ^de^ ± 3	22 ^ef^ ± 3	18 ^def^ ± 3	15 ^e^ ± 3
Cur-Cs/WPC NPs	31 ^a^ ± 4	22 ^ab^ ± 3	28 ^bcd^ ± 3	‘20 ^cde^ ± 3	23 ^bc^ ± 2

Different superscripts (a–g) in the same column are significantly different (*p* ˂ 0.05).
